# Accuracy of an automated system for tuberculosis detection on chest radiographs in high-risk screening

**DOI:** 10.5588/ijtld.17.0492

**Published:** 2018-05-01

**Authors:** J. Melendez, L. Hogeweg, C. I. Sánchez, R. H. H. M. Philipsen, R. W. Aldridge, A. C. Hayward, I. Abubakar, B. van Ginneken, A. Story

**Affiliations:** * Diagnostic Image Analysis Group, Radboud University Medical Center, Nijmegen; † Thirona, Nijmegen, The Netherlands; ‡ Department of Infectious Disease Informatics, Institute of Health Informatics, University College London, London; § Institute of Epidemiology and Health Care, University College London; ¶ Institute for Global Health, University College London, UK

**Keywords:** TB, computer-aided detection, chest radiography, computerised image analysis

## Abstract

**SETTING:**

Tuberculosis (TB) screening programmes can be optimised by reducing the number of chest radiographs (CXRs) requiring interpretation by human experts.

**OBJECTIVE:**

To evaluate the performance of computerised detection software in triaging CXRs in a high-throughput digital mobile TB screening programme.

**DESIGN:**

A retrospective evaluation of the software was performed on a database of 38 961 postero-anterior CXRs from unique individuals seen between 2005 and 2010, 87 of whom were diagnosed with TB. The software generated a TB likelihood score for each CXR. This score was compared with a reference standard for notified active pulmonary TB using receiver operating characteristic (ROC) curve and localisation ROC (LROC) curve analyses.

**RESULTS:**

On ROC curve analysis, software specificity was 55.71% (95%CI 55.21–56.20) and negative predictive value was 99.98% (95%CI 99.95–99.99), at a sensitivity of 95%. The area under the ROC curve was 0.90 (95%CI 0.86–0.93). Results of the LROC curve analysis were similar.

**CONCLUSION:**

The software could identify more than half of the normal images in a TB screening setting while maintaining high sensitivity, and may therefore be used for triage.

## Abstract (FR)

**CONTEXTE:**

Les programmes de dépistage de la tuberculose (TB) peuvent être optimisés en diminuant le nombre de radiographies pulmonaires (CXR) qui requièrent une interprétation par des experts.

**OBJECTIF:**

Evaluer la performance de logiciels de détection numérique pour le tri des CXR au sein d'un programme de dépistage de TB mobile numérique à haut débit.

**SCHÉMA:**

Une évaluation rétrospective du logiciel a été réalisée sur une base de données de 38 961 CXR de face réalisées entre 2005 et 2010 chez des patients dont 87 ont eu un diagnostic de TB. Le logiciel a généré un score de probabilité de TB pour chaque CXR. Ce score a été comparé à une norme de référence de TB pulmonaire active déclarée par analyse de courbe de la fonction d'efficacité du récepteur (ROC) et de localisation ROC (LROC).

**RÉSULTATS:**

Aprés analyse ROC, è une sensibilité de 95%, la spécificité a été de 55,71% (IC95% 55,21–56,20) et la valeur prédictive négative a été de 99,98% (IC95% 99,95–99,99). La zone sous la courbe ROC a été de 0,90 (IC95% 0,86–0,93). Les résultats après analyse LROC ont été similaires.

**CONCLUSION:**

Le logiciel peut identifier plus de la moitié des images normales dans un contexte de dépistage de la TB, tout en préservant la sensibilité élevée, et pourrait donc être utilisé pour le triage.

## Abstract (ES)

**MARCO DE REFERENCIA:**

Los programas de cribado de la tuberculosis (TB) se optimizan cuando se reduce el número de las radiografías de tórax (CXR) que exigen una interpretación por personas expertas.

**OBJETIVO:**

Evaluar la eficacia de un programa informético de detección que clasifique las CXR, en el marco de un programa de cribado de la TB informético ultrarrépido y portétil.

**MÉTODO:**

Se evaluó de manera retrospectiva el programa informético a partir de una base de datos de 38 961 CXR posteroanteriores realizadas del 2005 al 2010 en pacientes únicos, de los cuales en 87 se diagnosticó TB. El programa generaba una puntuacióon de probabilidad de TB en cada CXR. Esta puntuación se comparó con una norma de referencia de la TB pulmonar activa notificada, mediante un anélisis de rendimiento diagnóstico de la característica operativa del receptor (ROC) y un anélisis ROC de localización (LROC).

**RESULTADOS:**

Tras el anélisis ROC, con una sensibilidad de 95%, la especificidad fue 55,71% (IC95% 55,21–56,20) y el valor diagnóstico de un resultado negativo fue 99,98% (IC95% 99,95–99,99). El área bajo la curva de rendimiento diagnóstico fue 0,90 (IC95% 0,86–0,93). Los resultados del anélisis LROC fueron equivalentes.

**CONCLUSIÓN:**

El programa informético puede detectar més de la mitad de las CXR normales en el contexto del cribado de la TB y al mismo tiempo conserva una alta sensibilidad, por lo cual se puede utilizar en la selección radiogréfica.

WITH 10.4 MILLION NEW CASES and 1.8 million deaths in 2015, tuberculosis (TB) remains a major health concern. Prevalence is highest in Africa and overall incidence in Asia.^1^ Although TB incidence in the West has decreased, increases in TB rates have been reported in high-risk populations, especially in urban settings.^2^,^3^

Despite efforts to develop new TB diagnostics,^4–6^ screening is still commonly performed using chest radiography, followed by sputum culture, Xpert^™^ (Cepheid, Sunnyvale, CA, USA) testing or smear microscopy.^7^ Early studies reported limited specificity and variable levels of inter- and intra-reader agreement in interpreting chest radiographs (CXRs) for TB detection.^8^,^9^ However, modern digital radiography provides a quick and reliable technique with low marginal and operational costs,^10^ and its use, together with standardised scoring, may improve performance and reader agreement.^11–14^

Current screening programmes often require large volumes of CXRs to be manually assessed. The lack of skilled readers and their relatively high cost in some regions limit the potential of these programmes. In the present study, we proposed to examine the possibility of improving the efficiency of radiographic TB screening by introducing computer-aided detection (CAD) in the workflow. We determined the effect of applying CAD to triage individuals with suspected active pulmonary TB (PTB). In a screening context, triaging involves an initial triage test used to identify cases that require further investigation. These investigative tests typically have higher specificity, but also higher costs. In the proposed workflow (Figure 1), CXRs were analysed automatically directly after acquisition. The CAD system scored each image based on the likelihood of it containing TB-related abnormalities. Cases with a score below a cut-off value were considered normal, while cases above the cut-off value were subsequently examined by a human reader. The number of cases judged to be normal using CAD was equivalent to the reduction in the number of CXRs that needed to be read.

**Figure 1.**
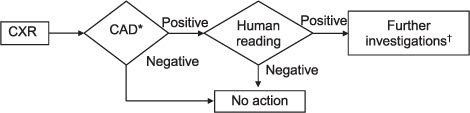
Proposed workflow with integration of CAD for tuberculosis detection as a first triage test before human reading.^*^ Predetermined cut-off value.^†^ Clinical and microbiological assessment. CXR = chest radiograph; CAD = computer-aided detection.

## STUDY POPULATION AND METHODS

### Ethics statement

This retrospective investigation was assessed by the Radboud University Medical Center, Nijmegen, The Netherlands, and the Health Research Authority of the National Health Service, London, UK (using the online tool at http://hra-decisiontools.org.uk/ethics/) to be an evaluation of an existing health care service using no identifiable patient information or possibility of deductive disclosure, and therefore not requiring ethical review.

### Study population

A large image database consisting of 47 510 postero-anterior CXRs from 39 328 individuals was created by the Find&Treat Screening Programme starting 1 April 2005 and ending 31 March 2010.^13^ The programme, organised in London, UK, screened a high-risk population of homeless people, prisoners and problem drug and alcohol users accessing homeless hostels, day centres, soup kitchens, drug treatment services and detention facilities. All individuals attending venues targeted by the mobile X-ray unit (Digital Diagnost, pixel spacing 0.143 mm, peak kilo voltage 90 kV; Philips Medical Systems, Amsterdam, The Netherlands) were eligible for screening. CXRs were read by a trained reporting radiographer. Notified cases of active PTB were defined as cases commenced on anti-tuberculosis treatment after radiological, clinical and microbiological investigations. In the United Kingdom, 30% of notified PTB cases are not culture-confirmed.^15^ As we were primarily concerned with the sensitivity of CAD for the purposes of triaging, and wished to identify early changes in paucibacillary cases using CAD, the clinical decision to treat, rather than culture confirmation, was used as a comparator.

The Find&Treat service provided an anonymised and de-duplicated set of 39 328 CXRs with data on TB status but no other clinical or identifier-related information. CXRs were categorised as ‘normal’, ‘abnormal but not active TB’ and ‘active TB’. For participants with repeat CXRs, only the most recent image was included, except when a CXR was associated with active TB, in which case that particular image was included.

### Computer-aided detection of tuberculosis on chest radiographs

The CAD software used in the study was CAD4TB 5 (Thirona, Nijmegen, The Netherlands), released in 2016, which uses a two-step pipeline to detect TB.

In the first step, a quality check component assesses whether the input is an appropriate CXR. Before quality assessment, energy-based normalisation is applied to reduce equipment-related differences among images.^16^ Valid CXRs continue to the next step.

In the second step, the TB analysis component starts by automatically segmenting unobscured lung fields.^17^ The goal is to restrict further analysis to these areas and to provide anatomical context for abnormality detection. As abnormalities are broadly defined as textural changes in the appearance of the lung parenchyma due to disease, a texture classifier is applied to highlight these changes. The output obtained is a heat map indicating the likelihood that a pixel belongs to an abnormal region. These pixel likelihoods are then summarised into a single score by applying a quantile rule.^18^ In addition to texture classification, the shapes of the segmented lung fields are also assessed. The rationale is that large abnormalities may affect this feature. The final CAD score is obtained by adding the scores for texture classification and shape analysis. For both lung segmentation and texture analysis, the software had been trained with manually annotated CXRs from various sources different from the one included in the present study.

### Evaluation procedure

The CAD scores generated for the CXRs in the data set were used to perform receiver operating characteristic (ROC) curve analysis compared with active TB references. To take into account the detection of actual radiological findings, localisation ROC (LROC) curve analysis was also carried out. For this purpose, lesions on CXRs of active TB cases were outlined and then scored by finding the highest pixel score in the lesion outlined. Finally, to ensure compatibility with the CAD4TB image scoring mechanism described in the previous subsection, the pixel score was clipped at the quantile value used by the software.

The CAD potential to triage was measured using the negative predictive value (NPV) and specificity at a score cut-off corresponding to 95% sensitivity. The overall performance of the CAD system was measured using the area under the curve (AUC). The NPV, specificity and AUC, with their 95% confidence intervals (CIs), were calculated using the ‘epiR’^19^ and ‘pROC’^20^ extension packages of R Software (R: A Language and Environment for Statistical Computing v3.3.1; R Foundation for Statistical Computing, Vienna, Austria).

## RESULTS

Of the 39 328 available CXRs, 367 could not be processed because of image data corruption; CAD scores were thus computed for 38 961 patients: 87 were active TB cases and 38 874 were ‘other’ cases, 37 288 of whom were normal and 1586 abnormal but not active TB cases. Of the 87 active TB cases, 70% (*n* = 61) were culture-confirmed; the remainder were determined to be active TB based on radiological and clinical investigations. Apart from the corrupted images mentioned above, the CAD software did not reject any CXR based on the output of its quality assessment component.

**Figure 2.**
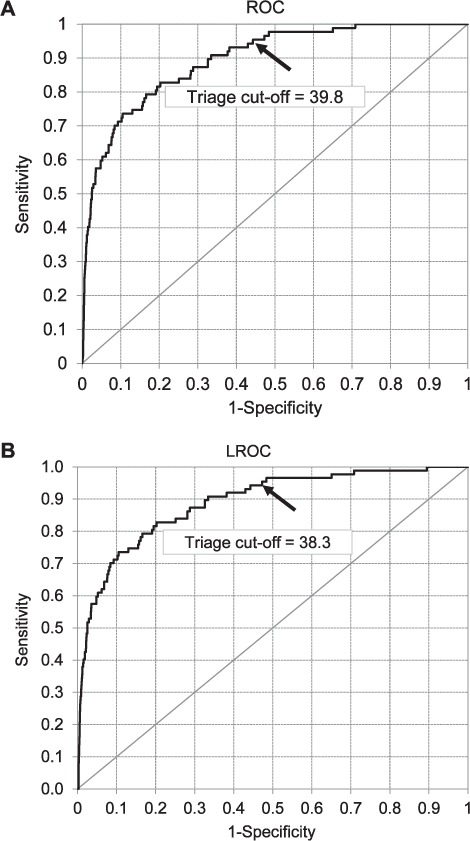
ROC and LROC curve analyses of CAD performance in discriminating between active TB and non-active TB cases. The triage cut-off point at 95% sensitivity is indicated by an arrow. Cases to the left of the cut-off point would be referred for human reading, whereas cases to the right would be excluded from further analysis. ROC = receiver operating characteristic; LROC = localisation ROC; CAD = computer-aided detection; TB = tuberculosis.

**Figure 3.**
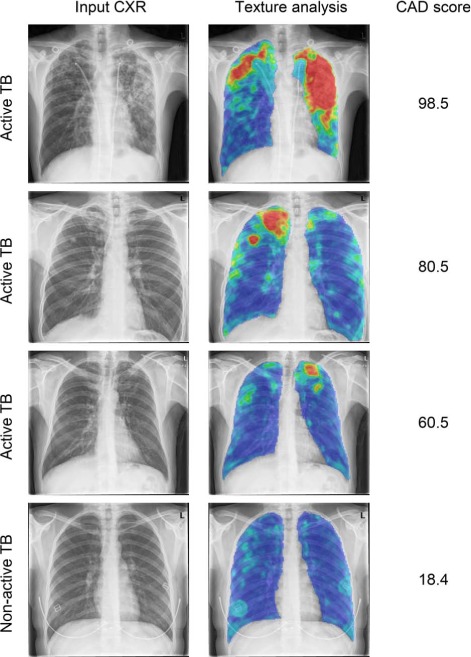
Examples of CAD analysis. First column: input CXR; second column: heat map resulting from texture analysis (colours indicate low-to-high suspicion of abnormality in the following order: blue-green-yellow-orange-red), last column: TB score assigned by CAD ranging from 0 (normal) to 100 (abnormal). CXR = chest radiograph; TB = tuberculosis; CAD = computer-aided detection. This image can be viewed online in colour at http://www.ingentaconnect.com/content/iuatld/ijtld/2018/00000022/00000005/art000...

After ROC curve analysis, the NPV was 99.98% (95%CI 99.95–99.99) and the AUC was 0.90 (95%CI 0.86–0.93). The score cut-off that led to the desired 95% sensitivity for triage was 39.8, and yielded a specificity of 55.71% (95%CI 55.21–56.20%). At this point, 21 656/38 874 other cases and 83/87 active TB cases were correctly identified. Detecting 100% of the active TB cases reduced specificity to 29.13% (95%CI 28.68–29.59). After LROC curve analysis, the NPV remained the same, while the specificity and the AUC decreased slightly to respectively 52.75% (95%CI 52.25–53.25) and 0.89 (95%CI 0.85–0.93). The triage cut-off point also decreased marginally to 38.3. The similarity between these two sets of results indicated that CAD4TB could provide not only good classification but also good lesion localisation. This may be verified by examining the ROC and LROC curves obtained (Figure 2). The output of the CAD system for a selected number of cases is shown in Figure 3.

## DISCUSSION

From our retrospective evaluation of CAD for radiological TB screening on a large database, we concluded that CAD could be used to exclude 55.71% of normal images from further reading, with a corresponding NPVof 99.98%, while maintaining a high sensitivity of 95%. The CAD system was tested on an unselected set of images obtained in a high-throughput screening setting targeting individuals at increased risk of TB. In such a setting, where the number of normal cases is typically much higher than the number of abnormal cases, the use of a CAD system may result in a large reduction of the workload for human readers of CXRs, thus substantially increasing screening cost-effectiveness.^21^

The sensitivity required for a test in a TB screening programme depends on the operational requirements of the setting in which it is deployed. For TB prevalence surveys, the World Health Organization (WHO) handbook recommends over-reading to reduce the chance of missing cases.^22^ Several performance measures of CXR for TB detection have been reported:^7^,^9^,^11^,^23–25^ sensitivities range from 25%^25^ to 95%^23^ and specificities from 53%^24^ to 99%.^25^ A high sensitivity of 95% was chosen for our study, as it equals the highest reported value in the literature. A sensitivity of 95% is also among the desired optimal and minimal characteristics for a TB triage test.^26^ At this sensitivity, the resulting specificity was 55.71%.

An important advantage of computerised reading is that all images are processed in a standardised, objective and repeatable way. This facilitates the integration of CXRs to a standardised screening protocol. A second advantage is that, unlike human readers, who typically provide binary scores, CAD produces a continuous score. This score can be used to set a specific cut-off point for different settings to meet particular operational requirements. A third advantage, in this case specific to CAD4TB, is that output is highly reliable, as it is driven by the localisation of actual lesions; this was confirmed by the similar results obtained with both ROC and LROC analyses. Moreover, the availability of a heat map highlighting suspicious image regions provides a perceivable explanation of the assigned TB score.

In high-burden, low-resource countries, where the availability of skilled CXR readers is limited, CAD could be used as the sole reader for screening.^27^ A cutoff point at high specificity but relatively low sensitivity could be used to reduce the number of normal cases receiving a follow-up test to make the final diagnosis. Furthermore, the use of CAD can increase the efficiency of national prevalence surveys. Prevalence surveys are recommended by the WHO to measure TB burden and the impact of TB control programmes;^28^ as these need to screen a large part of the population, cost reduction and high throughput are highly beneficial.^29^

A limitation of our study was that a relatively low percentage of the cases considered to be active TB cases were culture-confirmed (70%). However, there was a high level of follow-up after the treatment decision among presumptive TB cases by the outreach team. An additional strength of our study was evaluation of a large and highly representative sample in a real-world setting.

In low TB burden settings, where radiological screening is mainly employed for high-risk groups and migrants from endemic countries, CAD can be used as a first reader, and cases marked as ‘suspected TB’ using CAD can then be assessed by a human reader. The fractional reduction in the workload in this scenario is almost directly proportional to the specificity, as the percentage of active TB cases is generally small. For example, had CAD been used prospectively to triage active TB cases on the full Find&Treat database consisting of 47 510 CXRs, the number of cases not referred to the human reader would have been reduced to 26 467, i.e., 55.71% of the total number. Alternatively, CAD could be employed as a second reader for quality assurance, for example in pre- and post-entry screening programmes. Given that the performance of the software evaluated in our study was comparable with that of expert readers (e.g., radiologists),^29–31^ such applications seem feasible.

## CONCLUSION

CAD can be used to identify a large proportion of normal CXRs in a TB screening setting at high sensitivity, and could therefore be an instrument of triage. This could increase the cost-effectiveness of radiographic TB screening. Future work should focus on further increasing CAD specificity and prospective evaluation in screening programmes.
